# Trophoblast Extracellular Vesicles as Modulators of Keratinocyte Stress Response and Senescence

**DOI:** 10.3390/life15060918

**Published:** 2025-06-05

**Authors:** Mirjana Nacka-Aleksić, Andrea Pirković, Aleksandra Vilotić, Maja Kosanović, Dragana Dekanski, Janko Legner, Milica Jovanović Krivokuća

**Affiliations:** 1 University of Belgrade, Institute for the Application of Nuclear Energy (INEP), Department for Biology of Reproduction, Banatska 31b, 11080 Zemun, Serbia; andrea.pirkovic@inep.co.rs (A.P.); dragana.dekanski@inep.ac.rs (D.D.); janko.legner@inep.co.rs (J.L.); milicaj@inep.co.rs (M.J.K.); 2 University of Belgrade, Institute for the Application of Nuclear Energy (INEP), Department for Immunology and Immunoparasitology, Banatska 31b, 11080 Zemun, Serbia

**Keywords:** skin, aging, placenta, wound healing, regenerative medicine

## Abstract

Keratinocyte stress, caused by various intrinsic and extrinsic factors, contributes to the overall aging process. D-galactose-induced metabolic/oxidative stress is a commonly used *in vitro* model for studying premature aging. Due to their rich composition of bioactive molecules that influence critical pathways in cellular aging and rejuvenation, placental derivatives have a well-established history in anti-aging skincare and therapy. However, trophoblast-derived extracellular vesicle (TEV) effects on D-galactose-induced premature aging in keratinocytes have not been investigated yet. TEV pretreatment for 24 h enhanced cellular resilience against D-galactose-induced stress, judging by the downregulated expression of senescence- and stress-associated markers (p19 and p21, HIF-1α, mTOR), and reduced production of reactive oxygen species and DNA damage. Additionally, TEV pretreatment enhanced keratinocyte proliferation and integrin-β1 subunit expression upon D-galactose exposure, most likely contributing to more efficient wound closure. In conclusion, this study underscores the potential of TEVs to modify expression of stress- and senescence-related proteins in keratinocytes and improve their wound healing properties. Their regenerative and protective characteristics position TEVs as promising candidates for developing innovative procedures to address skin conditions related to premature aging.

## 1. Introduction

Keratinocytes represent 95% of skin epidermal cells and play an integral role in skin homeostasis and barrier function [1]. In addition to the genetic, hormonal, and metabolic drivers of intrinsic aging, extrinsic factors such as ultraviolet (UV) radiation, air pollution, “unhealthy” lifestyles, and continuous wear-and-tear processes significantly contribute to keratinocyte senescence [2]. This state is characterized by a stable cell cycle arrest, impaired DNA repair mechanisms, decreased proliferative capacity, an inflammatory phenotype and altered secretory profiles, leading to compromised skin integrity and barrier function [3].

Numerous studies have shown that increased dietary carbohydrate intake is linked to skin aging [4]. Carbohydrates can damage essential skin components like elastin and collagen through non enzymatic protein glycation, leading to the formation of advanced glycation end-products (AGEs) [5]. This process is closely associated with oxidative stress (OS) [6]. D-galactose (D-gal) is a more potent oxidant and glycation agent than glucose [7]. Free galactose is found in various fruits and vegetables, including tomatoes, brussels sprouts, bananas, and apples [8,9]. Lactose hydrolysate syrup, which contains high levels of galactose, is commonly used in baked and dairy products [9]. D-gal is widely used in experimental models to induce metabolic stress and aging-like phenotypes in cells [6,10]. When administered at elevated levels, D-gal leads to OS and mitochondrial dysfunction, triggering a cascade of events resulting in cell cycle arrest, altered gene expression, inflammation, and cellular dysfunction [6]. Significant morphological changes in the skin of rodents treated with D-gal have also been observed, including skin thinning and deterioration of fur quality [11,12,13]. Accumulation of senescent cells in the skin leads to reduced cell migration, changes in extracellular matrix (ECM) composition, and ultimately contributes to common signs of skin aging such as loss of elasticity and wrinkles [14].

Historically, the placenta and its derivatives have a rich precedent in the field of anti-aging skin care and therapy [15]. Nanosized extracellular vesicles (EVs) are released from all cells into their extracellular environment, carrying different types of biomolecules to target cells, affecting their physiology [16]. Trophoblast-derived EVs (TEVs) play pivotal roles in intercellular communication and maintenance of immune tolerance at the fetal–maternal interface by transferring proteins/peptides, nucleic acids, vitamins, trace elements, growth factors, lipids, and other bioactive molecules [17]. Thereby, TEVs may regulate numerous aspects of recipient cell phenotype and function, including cell cycle, migration, synthesis of inflammatory mediators, and pathways involved in cellular aging and/or rejuvenation [18]. Placental and partially purified EVs released from first-trimester extravillous trophoblast (EVT) cell line Swan 71 were shown to contain heat shock proteins, cytoskeletal proteins, adhesion and fusion proteins, histones, enzymes involved in amino acid and lipid metabolism, redox reactions, protein synthesis, DNA replication, mRNA splicing, transcription, and translation, as well as placenta-specific proteins (pregnancy zone protein and human chorionic gonadotropin) [19,20]. Additionally, mass spectrometry analysis of first-trimester EVT cell line HTR-8/SVneo-derived EVs identified over 140 proteins associated with cellular movement and morphology, immune cell trafficking, and cellular assembly and organization [21]. Our group has previously shown that TEVs also contain galectins 1, 3, and 8 [22], which are known to be involved in keratinocyte adhesion, migration, differentiation, proliferation, and apoptosis, as critical processes in skin health and wound healing [23]. Other studies have also demonstrated TEV influence on immune responses, angiogenesis, and tissue remodeling in both physiological and pathological contexts [20,24,25,26]. However, their potential to combat keratinocyte stress and related senescence have not been investigated before. Therefore, the study herein aimed to investigate the effects of TEVs released by first-trimester EVT HTR-8/SVneo cell line on D-gal-induced OS/metabolic stress in human keratinocytes, and shed light on potential implications for skin care, regenerative, and anti-aging strategies.

## 2. Materials and Methods

### 2.1. Cell Cultures

Spontaneously immortalized human keratinocyte cell line HaCaT (a generous gift from Dr. Milica Pešić, Institute for Biological Research “Siniša Stanković”, University of Belgrade, Serbia) was used to model keratinocyte functions [27,28]. HaCaT cells were cultured in Dulbecco’s Modified Eagle Medium (DMEM)-F12 supplemented with 10% fetal bovine serum (FBS) and 1% antibiotic/antimycotic solution (herein: complete culture medium). HTR-8/SVneo immortalized human first-trimester EVT cell line (kindly provided by Dr. Charles H. Graham, Queen’s, Kingston, ON, Canada) was cultured in RPMI 1640 medium, supplemented with 10% FBS and 1% antibiotic/antimycotic solution (Capricorn Scientific GmbH, Ebsdorfergrund, Germany). Cell culture media and FBS were obtained from Biowest, Nuaillé, France.

### 2.2. TEV Enrichment

TEVs were enriched from HTR-8/SVneo cell-conditioned medium by ultracentrifugation [22]. Briefly: 5 × 10^6^ HTR-8/SVneo cells were cultured in T175 flasks until they reached approximately 80% confluence. After washing the cells twice with 0.05 M PBS (pH 7.2), they were maintained in serum-free medium for an additional 24 h. Then, the conditioned medium was collected and TEVs were enriched by differential centrifugation: cells and debris were removed at 300× *g* for 15 min, followed by 3000× *g* for 20 min and 16,500× *g* for 25 min. The final supernatant was gently passed through a 0.22 μm filter and subjected to ultracentrifugation at 80,000× *g* (k factor 262) for 2 h at 4 °C to collect the pellet enriched in TEVs (Optima L-90K ultracentrifuge with a Ti 50.2 rotor, Beckman Coulter, Brea, CA, USA). The total protein content in the TEV-enriched preparation (hereafter TEVs) was quantified using the Pierce Bicinchoninic Acid (BCA) Protein Assay Kit from Thermo Fisher Scientific (Waltham, MA, USA).

### 2.3. Nanoparticle Tracking Analysis (NTA)

TEV number and size were assessed by NTA, using ZetaView Quatt PMX-430 with ZetaView software version 8.05.16 SP3 (Particle Metrix, Inning, Germany). Automatic cell check, alignment of camera and laser, and focus optimization were performed according to manufacturer’s instructions, using 100 nm polystyrene beads. Samples were diluted (1:250) in PBS to fit the number of particles in the measurement range (50–200). Blue laser (488 nm) was used to illuminate the particles in suspension and the measurements were performed in scatter mode. The video was captured at a sensitivity of 78, a shutter speed of 100, and a frame rate of 30 frames/s, during one cycle. Post-acquisition parameters were set to a minimal area 10, maximal area 1000 and minimum brightness 30. Samples were measured at 11 positions to determine median size.

### 2.4. Transmission Electron Microscopy (TEM)

Samples of enriched TEV preparations were analyzed by TEM in order to confirm their presence and assess their morphology. Briefly, copper grids (200 mesh, coated with Formvar) were floated on droplets of the TEV-enriched preparation, for 45 min; meshes were then placed on droplets of 2% paraformaldehyde for 10 min and rinsed by placement on droplets of PBS, 3 times for 2 min. Subsequently, meshes were placed on droplets of 2.5% glutaraldehyde for 5 min and on dH_2_O droplets for 2 min. Excess fluid was removed between each step. All incubations were carried out in a humid chamber at room temperature and all droplets were of 10 μL. After the final step, the grids were allowed to air dry. Electron micrographs were collected using a Philips CM12 electron microscope (Philips, Eindhoven, The Netherlands) equipped with the digital camera SIS MegaView III and iTEM software (Olympus Soft Imaging Solutions, Münster, Germany).

### 2.5. TEV Super-Resolution Imaging

TEV samples were processed using the Application Kit™ EV Profiler 2 (Cat. no. 900-00079, ONI, Oxford, UK) and imaged through direct Stochastic Optical Reconstruction Microscopy (dSTORM) on the Nanoimager S Mark III microscope (ONI, Oxford, UK), operated by an ONI technical expert. dSTORM, a single-molecule localization technique, offers a resolution of 20 nm. TEV samples were prepared according to the manufacturer’s protocol. Affinity capture of TEVs was performed on a 4-lane chip using biotinylated Tetraspanin Trio (anti-CD81/anti-CD63/anti-CD9) for capture. Following a 10 min fixation, the TEVs were labeled with anti-CD81-AlexaFLuor 647, anti-CD63-AlexaFLuor 561, and anti-CD9-AlexaFLuor 488 (ONI, Oxford, UK), each conjugated to dSTORM-compatible dyes provided in the kit. TEVs were imaged on the Nanoimager (ONI, Oxford, UK) using a 1.45 NA 100× oil immersion objective. The system was calibrated with a bead slide prior to sample imaging. TIRF illumination was set at a 54.1° angle, and the fluorescent dyes were excited with 170 mW (640), 44 mW (561), and 180 mW (488) laser powers. Image acquisition involved 30 ms exposure time and 1000 frames per channel. Six fields of view (FOVs) were recorded for each sample. Image processing and quantification were performed using ONI’s online software platform (CODI), with drift correction applied. dSTORM filtering was applied for frame index (640: 50–999, 561: 1050–1999, 488: 2050–2999), photon count (200–7500), sigma (50–225 nm), and localization precision (0–10 nm). A density-based clustering method was used, with TEVs identified as positive based on having three or more localizations per channel.

### 2.6. Experimental Design

The working concentration of D-gal (Sigma-Aldrich, St. Louis, MO, USA) was chosen based on MTT (3-(4,5-dimethylthiazol-2-yl)-2,5-diphenyltetrazolium bromide) assay with serial dilutions of D-gal, as the highest concentration of D-gal that does not affect cell viability (Figure S1). Briefly: Keratinocytes (2 × 10^4^/well) were seeded in 96-well plates in 100 µL of complete medium and allowed to adhere overnight. After washing with PBS, the cells were further cultured 24 h in complete medium with or without D-gal (20, 40, 60, 80 mg/mL). MTT in the final concentration of 0.5 mg/mL was added and incubated for 3 h at 37 °C, 5% CO_2_. Subsequently, 100 μL of 10% SDS (0.01 N HCl) was added and incubated overnight. The absorbance was measured at 570 nm using a microplate reader (Agilent BioTek Epoch, Santa Clara, CA, USA).

In the subsequent experiments, keratinocytes were seeded at a density of 1 × 10^5^ cells per well in 96-well plates (or in 24-well plates, respectively) and cultured overnight to adhere. For keratinocyte pretreatment 50 µg/mL of TEV protein were used, as it has been shown that concentrations around 50 µg/mL of EV protein are beneficial for promoting cell migration, proliferation, and tissue regeneration in wound healing [29,30]. Based on NTA, the 50 µg/mL TEV protein concentration corresponded to an approximate of 4.4 × 10^8^ particles/mL, yielding an estimated TEV-to-cell ratio of approximately 4000:1.

Keratinocytes were pretreated with TEVs for 24 h and then exposed to 20 mg/mL of D-gal for 48 h to induce cellular stress leading to senescence. The 48 h treatment window is a well-established timeframe for triggering senescence *in vitro*, particularly in models using D-gal [31,32]. During this period, cells experience significant metabolic changes, including DNA damage accumulation, reactive oxygen species (ROS) production, and changes in cell signaling pathways that lead to senescence [31,32]. This timeframe also allows for the establishment of stable cellular senescence without excessive cell death, ensuring enough cells and sufficient cell viability for downstream analyses of molecular mechanisms.

### 2.7. Cell-Based ELISA Assay (cELISA)

Protein expression in keratinocytes was determined by cELISA. Briefly, after fixing the cells with ice-cold acetone/methanol (1:1) for 5 min and blocking with 1% bovine serum albumin (BSA) in PBS for 30 min, they were incubated with rabbit polyclonal anti-p19^ARF^ (hereafter: p19), anti-p21^CIP1/WAF1^ (hereafter: p21), anti-hypoxia-inducible factor (HIF)-1α (all Santa Cruz Biotechnology, Inc., Dallas, TX, USA), anti-integrin-β1(Merck KGaA, Darmstadt, Germany), and anti-Ki-67 (Sigma Aldrich, St. Louis, MO, USA) antibodies, diluted in 1% BSA/PBS at 4 °C overnight in a humidified chamber. For assessment of non-specific binding, cells were incubated in 1% BSA/PBS. Cells were rinsed with PBS and incubated with appropriate horseradish peroxidase (HRP)-conjugated secondary goat anti-rabbit IgG (Cell Signaling Technology, Danvers, MA, USA), diluted in 1% BSA/PBS for 1 h at room temperature in the dark. Following incubation and rinsing with PBS, 50 µL/well tetramethylbenzidine substrate was added. The reaction was stopped with 0.2 M H_2_SO_4_ (50 µL/well). Absorbance was read at 450 nm (Agilent BioTek Epoch, Santa Clara, CA, USA). The absorbance for non-specific binding was subtracted from all absorbances.

### 2.8. 2′,7′–Dichlorofluorescin Diacetate (DCFH-DA) Assay

To quantitatively determine ROS in keratinocytes, DCFH-DA was used as a fluorescent oxidation-sensitive probe (Merck KGaA, Darmstadt, Germany). This reagent is cleaved by intracellular esterase enzymes, resulting in formation of fluorescent 2,7-dichlorofluorescein. The intensity of fluorescence is directly proportional to the ROS levels. For this experiment, samples were mixed with 2 mM H2DCFDA and incubated in amber tubes in the dark at room temperature for 40 min. Following incubation, 2 mL of PBS was added and 200 μL of each sample was added to a 96-well plate (PBS was used as blank). ROS level was determined by measuring the fluorescence on a fluorescent plate reader Victor3V (PerkinElmer, Boston, MA, USA) at excitation and emission wavelengths of 485 nm and 535 nm, respectively.

### 2.9. Senescence-Associated β-Galactosidase (SA-β-Gal) Staining

To assess SA-β-gal expression in HaCaT cells, a Cellular Senescence Assay Kit (Sigma-Aldrich, St. Louis, MO, USA) was used. In brief, upon pretreatment with TEVs for 24 h and exposure to D-gal for 48 h, keratinocytes were washed with 1× PBS and fixed using a 1× Fixing Solution (diluted 100× Fixing Solution with PBS). After fixation for 10–15 min at room temperature, cells were washed twice with PBS. A freshly prepared SA-β-Gal Detection Solution was then added, comprising Staining Solutions A and B, X-Gal, and PBS, mixed according to the manufacturer instructions. Cells were incubated with the detection solution at 37 °C (without CO_2_) for a minimum of 4 h, protected from light. Post-incubation, the development of a blue color, which should indicate SA-β-Gal activity, was assessed under a light microscope.

### 2.10. Alkaline Comet Assay

TEVs were added to keratinocytes for 24 h and then the cells were stressed by exposing them to D-gal for 48 h. After collecting, cells were centrifuged at 300× *g* for 5 min, and the obtained pellet was used for the comet assay. Briefly: The single-cell suspension at 1 × 10^4^/mL density was mixed with 0.7% low-melting-point agarose (Sigma Aldrich, St. Louis, MO, USA) and spread on previously pre-coated slides (with 1% normal-melting-point agarose, Sigma Aldrich, St. Louis, MO, USA). After the gel solidified, slides were placed in lysing solution, and a lysis step was then followed by DNA unwinding and electrophoresis (run at 25 V and 300 mA for 30 min). The slides were neutralized with PBS and the staining was performed with ethidium bromide (20 µg/mL). Comets were scored at a magnification of 40× Olympus IX73 inverted microscope (Olympus Optical Co., GmbH, Hamburg, Germany) equipped with pE-3000^white^ illuminator (CoolLED Ltd., Andover, UK) and Olympus SC50 digital camera (Olympus Optical Co., GmbH, Germany). Visual scoring and classification of the DNA damage: Each of the observed comets was assigned to a class of A, B, C, D, or E according to the amount of fragmented DNA in the comet tail: (A) no damage: <5%; (B) low-level damage: 5–20%; (C) medium-level damage: 20–40%; (D) high-level damage: 40–75%; (E) total damage: >75%. To semi-quantitatively analyze the data, DNA damage was characterized as DNA migration over 5% (sum of comet classes B + C + D + E). Two replicate slides were analyzed for each treatment, and the scoring was performed on 100 randomly selected comets per slide. Results are presented as the percentage of cells with DNA damage (B + C + D + E categories) per 100 nucleoids.

### 2.11. Flow Cytometry

For cell cycle analysis, keratinocytes, previously treated with TEVs for 24 h and then exposed to D-gal for 48 h, were detached using trypsin (Biowest, Bradenton, FL, USA)–EDTA (Sigma Aldrich, St. Louis, MO, USA) and washed twice with PBS. The collected cells were resuspended in 200 μL of PBS, and 3 mL of ice-cold 70% ethanol was slowly added to fix the cells for 1 h at 4 °C, followed by two PBS washes. Finally, 500 μL of propidium iodide solution was added, and cell cycle distribution was analyzed using a BD^®^ LSR II Flow Cytometer (Becton Dickinson, Mountain View, CA, USA) and FlowJo software v10.10. (TreeStar Inc., Ashland, OR, USA).

### 2.12. qPCR Analysis

Keratinocytes were seeded in 24-well plates and left to adhere overnight, followed by 24 h incubation with TEVs. Next, D-gal was added and after 48 h, and the cells were rinsed with PBS and lysed in TRI Reagent solution. Total RNA was isolated by manufacturer instructions and first-strand cDNA was synthesized from 1 μg of total RNA, using 0.5 μg of Oligo(dT) 12–18 primers, 250 μM of each dNTP, and 200 U of RevertAid reverse transcriptase (all reagents from Thermo Fisher Scientific Baltics, Vilnius, Lithuania). Real-time PCR was performed using a 7500 Real-Time PCR System (Applied Biosystems, Foster City, CA, USA). The reaction mixture contained 1 μL of cDNA, 5 μL of 2× SYBR™ Green PCR Master Mix (Applied Biosystems, USA), and specific forward and reverse primer in a final concentration of 0.5 μM. Reactions were run at 95 °C for 10 min, followed by 40 cycles of 15 sec at 95 °C and 1 min at 60 °C. Melting curve analysis was performed to verify amplification specificity. Expression levels of the mammalian target of rapamycin (mTOR) gene *MTOR* and β-galactosidase gene *GLB1* were normalized to the housekeeping gene *GAPDH*. Relative gene expression was calculated by the 2^−ΔΔCt^ method.

Gene primer sequence:

*MTOR* Forward: 5′-AGCATCGGATGCTTAGGAGTGG-3′

*MTOR* Reverse: 5′-CAGCCAGTCATCTTTGGAGACC-3′

*GLB1* Forward: 5′-CACTCCACAATCAAGACCGAAGC-3′

*GLB1* Reverse: 5′-CTGTGCTGCATAGGGTGAGTTG-3′

*GAPDH* Forward: 5′-GAAGGTGAAGGTCGGAGT-3′

*GAPDH* Reverse: 5′-GAAGATGGTGATGGGATTTC-3′

### 2.13. Wound Healing Assay

The influence of TEVs on D-gal-stressed keratinocyte migration was evaluated using a classic scratch (“wound healing”) assay [33]: the cells, pretreated with TEVs for 24 h and exposed to D-gal for 48 h, were scratched using a 200 μL sterile pipette tip and rinsed with PBS twice to remove detached cells. The pre-selected fields were photographed at 0 h and 24 h and the area of the scratch was measured at each time point. The images acquired for each sample were analyzed quantitatively by using the Wound Healing Tool (RRID:SCR_025260) for ImageJ v1.54k. Wound area reduction was calculated as the percentage of closure relative to the initial scratch area.

### 2.14. Statistical Analysis

Data was analyzed using one-way analysis of variance (ANOVA) with Tukey post hoc test (GraphPad Software, Inc., Boston, MA, USA). Values were considered significantly different when *p* < 0.05. The number of experiments is given in each figure legend.

## 3. Results

### 3.1. TEV Characterization

**Figure 1:**
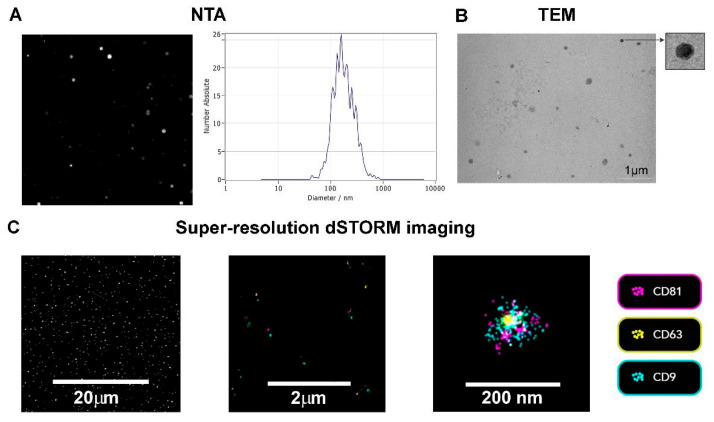
Characterization of trophoblast-derived extracellular vesicles (TEVs) by nanoparticle tracking analysis (NTA), transmission electron microscopy (TEM), and super-resolution dSTORM imaging. (**A**) Representative frame from ZetaView Quatt PMX-430 showing concentration and size measurement of TEVs by NTA. (**B**) Representative TEM native image of TEVs (inset displays zoomed-in vesicle). (**C**) Representative ONI super-resolution nanoimaging TEV captures. Antibodies targeted against CD9-AlexaFluor™ 488 (cyan), CD63-AlexaFluor™ 561 (yellow), and CD81-AlexaFluor™ 647 (purple) allowed for detection of their surface expression of immobilized TEVs (see Materials and Methods).

TEV preparations enriched by differential ultracentrifugation were analyzed for size and concentration of the particles, and the expression of classical EV markers, i.e., tetraspanins CD63, CD9, and CD81 [34]. NTA revealed a heterogeneous population of particles, ranging between 111 and 302 nm, with a mean size of 167.9 nm and concentration of 1.2 × 10^10^ particles/mL (Figure 1A).

To better characterize the vesicles at a single-EV level, TEV morphology and co-localization of tetraspanins were assessed using TEM and super-resolution dSTORM microscopy. TEM confirmed the presence of electron dense structures, characteristic for EVs, with preserved integrity (Figure 1B), while the super-resolution imaging confirmed the expression of EV markers on TEV surface (Figure 1C). According to CODI analysis, 39% of the TEVs were triple positive, whereas 92.5%, 51.8%, and 78.9% of TEVs were single positive for CD9, CD63, or CD81, respectively.

### 3.2. TEV Pretreatment Alleviates D-Gal-Induced OS and DNA Damage in Keratinocytes

**Figure 2:**
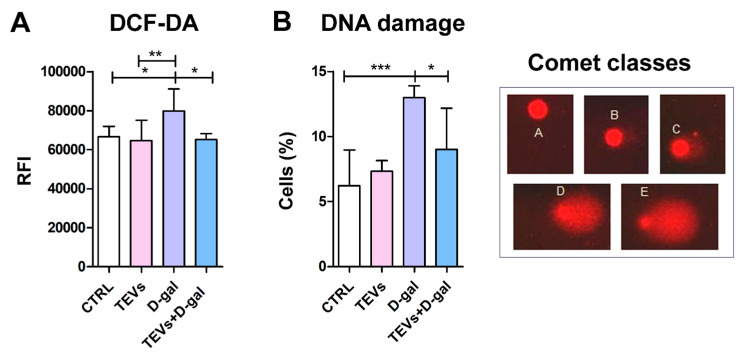
Trophoblast-derived extracellular vesicle (TEV) pretreatment diminishes reactive oxygen species (ROS) level and DNA damage in keratinocytes exposed to D-gal. Bar graphs indicate (**A**) relative fluorescence intensity (RFI) of dihydrofluorescein dye (DCF-DA) in keratinocytes, which correlates with intracellular ROS levels, and (**B**) percentage of cells with DNA damage, assessed by the alkaline comet assay. Representative microscopic images (40× magnification) show keratinocyte nuclei with damaged DNA (comets). Visual scoring of the comets to five classes according to the amount of fragmented DNA in the comet tail: A. no damage, <5%; B. low-level damage, 5–20%; C. medium-level damage, 20–40%; D. high-level damage, 40–75%; E. total damage, >75%. Cumulative data from two experiments (3–4 replicates) are expressed as mean + SD. * *p* < 0.05; ** *p* < 0.01; *** *p* < 0.001.

D-gal is a monosaccharide that has been widely used in experimental models to induce senescence, characterized by OS, inflammation, and cellular dysfunction. Chronic exposure to D-gal may overwhelm cellular antioxidant defenses, reducing cell capacity to neutralize ROS thereby leading to oxidative injury [6,10]. Expectedly, D-gal exposure significantly increased ROS levels in HaCaT cells after 48 h of incubation (Figure 2A). On the other hand, the level of ROS in the keratinocytes pretreated with TEVs and then exposed to D-gal was similar as in the control cells, whereas TEVs alone did not induce any change in the level of ROS in the cells (Figure 2A).

One major consequence of OS is DNA damage, including base and/or sugar alterations, sugar–base cyclization, DNA–protein cross-links, and intra- and interstrand cross-links, which in turn can result in DNA strand breaks [35]. To evaluate the capacity of TEVs to protect keratinocytes from D-gal-induced genotoxic stress, we employed the alkaline comet assay, a gel electrophoresis-based method to measure DNA damage, including single- and double-strand DNA breaks in individual eukaryotic cells [36]. D-gal after 48 h markedly increased the frequency of cells with DNA damage compared to control keratinocytes and cells treated with TEVs alone (Figure 2B). TEV-pretreated keratinocytes showed significantly lower frequency of cells with DNA damage following D-gal exposure, suggesting TEV genoprotective properties that help keratinocytes resist OS (Figure 2B).

In addition, OS is known to trigger the expression of SA-β-Gal, widely used as a marker of cellular senescence. Its activity is driven by the lysosomal β-galactosidase enzyme encoded by the *GLB1* gene, the expression of which is not universal across all cell types undergoing senescence [37]. Previously, SA-β-Gal activity was shown to be absent or markedly reduced in HaCaT cells, even under stress conditions such as H_2_O_2_ exposure, likely due to their unique genetic or epigenetic characteristics [38].

To explore this further, we analyzed both *GLB1* mRNA expression and SA-β-Gal staining in our HaCaT cells. The results showed no significant change in *GLB1* expression, and SA-β-Gal activity remained undetectable after D-gal exposure (Figure S2).

This finding is consistent with reports in some other cell types, including certain epithelial cells and murine fibroblasts, which often display weak or no SA-β-Gal staining despite exhibiting other hallmarks of senescence [39,40,41], including downregulation of Klotho protein [38] and upregulation of tumor suppressor proteins, as observed in our study. Positive SA-β-Gal staining has also been observed in non-senescent contexts, such as chemically induced differentiation of human cancer cells or during contact inhibition [39,40,41]. This suggests that, despite being broadly considered a marker of cellular senescence, the specificity and reliability of SA-β-Gal activity for accurately identifying senescent cells remain questionable.

### 3.3. TEV Pretreatment Mitigates D-Gal-Induced Upregulation of Senescence Markers in Keratinocytes

**Figure 3:**
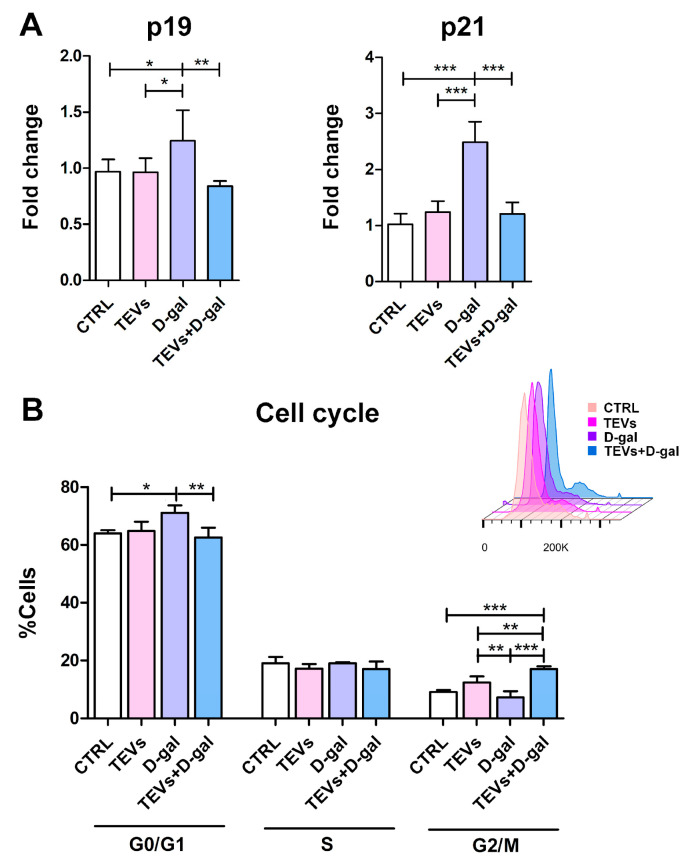
Pretreatment of keratinocytes with trophoblast-derived extracellular vesicles (TEVs) prevents upregulation of senescence markers and cell cycle arrest. Bar graphs indications: (**A**) Expression of cyclin-dependent kinase inhibitors p19 and p21 in keratinocytes, as determined by cELISA. Cumulative data from two experiments (3 replicates) are shown as mean fold change relative to the unexposed cells (CTRL) + SD. (**B**) Representative overlaid flow cytometry histograms indicate the frequency of cells in G0/G1, S, G2/M phases of the cell cycle. Data (mean + SD) are representative from one of two experiments (4 replicates) with consistent results. * *p* < 0.05; ** *p* < 0.01; *** *p* < 0.001.

Accumulation of DNA damage due to OS activates DNA damage response pathways, including the p19–p53 pathway and its downstream target, p21, which inhibit the cyclin-dependent kinase (CDK)-dependent phosphorylation of Rb (retinoblastoma protein), leading to cell cycle arrest at the G0/G1 phase [42]. This prevents further multiplication of damaged cells, where the cells remain metabolically active but no longer proliferate [43]. D-gal exposure induced upregulation of CDK inhibitors p19 and p21, indicating that the keratinocytes are undergoing cellular stress leading to senescence. On the other hand, TEV pretreatment considerably prevented this effect, while TEVs alone did not induce a significant change in the protein level of these senescence markers (Figure 3A).

The previous findings were further supported by flow cytometry cell cycle analysis: most of the untreated control cells were distributed in the G0/G1 phase, with a moderate proportion in the S and G2/M phases, consistent with normal proliferative activity. Exposure to D-gal increased the percentage of cells in the G0/G1 phase, accompanied by a corresponding reduction in the G2/M phase population (Figure 3B). This shift suggests a D-gal-induced G1 cell cycle arrest, indicative of reduced DNA synthesis and proliferative capacity. In contrast, TEV pretreatment showed a partial reversal of the D-gal effect, with a decreased frequency of cells in the G0/G1 phase and an increased prevalence of cells in the G2/M phase (Figure 3B). This suggests that TEVs may mitigate D-gal-induced cell cycle disruption and support the proliferative state of HaCaT cells.

### 3.4. TEV Pretreatment Downregulates HIF-1α and mTOR Expression in D-Gal-Exposed Keratinocytes

**Figure 4:**
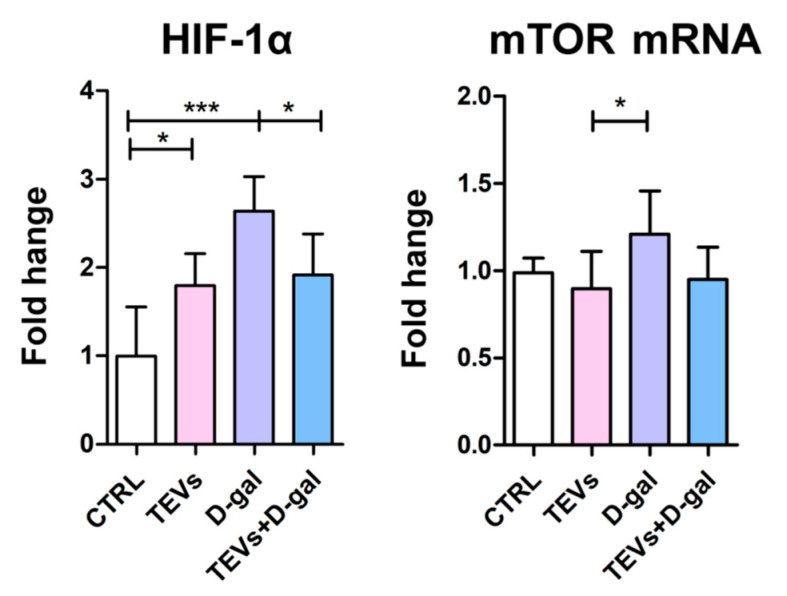
Trophoblast-derived extracellular vesicle (TEV) pretreatment ameliorates HIF-1α and mTOR upregulation in D-gal-exposed keratinocytes. Bar graphs indicate protein expression of HIF-1α and mRNA expression of mTOR in keratinocytes, as determined by cELISA and qPCR, respectively. Cumulative data from two experiments (3 replicates) are shown as mean fold change relative to the unexposed cells (CTRL) + SD. * *p* < 0.05; *** *p* < 0.001.

Considering that HIF-1α (i) accumulates in hypoxic or otherwise stressed cells and induces cellular senescence by promoting the expression of p21 and other senescence markers [44] and (ii) is a critical regulator of skin homeostasis with a pivotal role in epidermal aging and wound healing [45], its expression was also investigated. HIF-1α protein level increased in the cells exposed to either TEVs or D-gal alone, indicating an adaptive response to maintain cellular homeostasis; however, prolonged activation of this pathway can contribute to inflammation and senescence [46]. When the keratinocytes were preincubated with TEVs for 24 h prior D-gal exposure, HIF-1α levels were significantly lower compared to those in the cells exposed to D-gal only (Figure 4), suggesting TEV pretreatment could prevent the accumulation of HIF-1α upon D-gal exposure and restore metabolic balance.

HIF-1α activity is also modulated by mTOR [47], a kinase implicated in cellular metabolism, growth, and aging [48]; thus, its gene expression was also measured. The mRNA expression of mTOR increased after D-gal exposure, though this increase was significant only compared to the cells treated with TEVs alone (Figure 4). This was consistent with previous reports of upregulated mTOR expression in keratinocytes after D-gal administration [42], likely in response to D-gal-induced OS [49]. Interestingly, TEVs alone decreased mTOR mRNA expression compared to controls, whereas TEV pretreatment combined with D-gal exposure maintained mTOR levels close to the control ones (Figure 4).

### 3.5. TEV Pretreatment Increases Keratinocyte Proliferation Rate, Migration, and Integrin-β1 Subunit Expression After D-Gal Exposure

**Figure 5:**
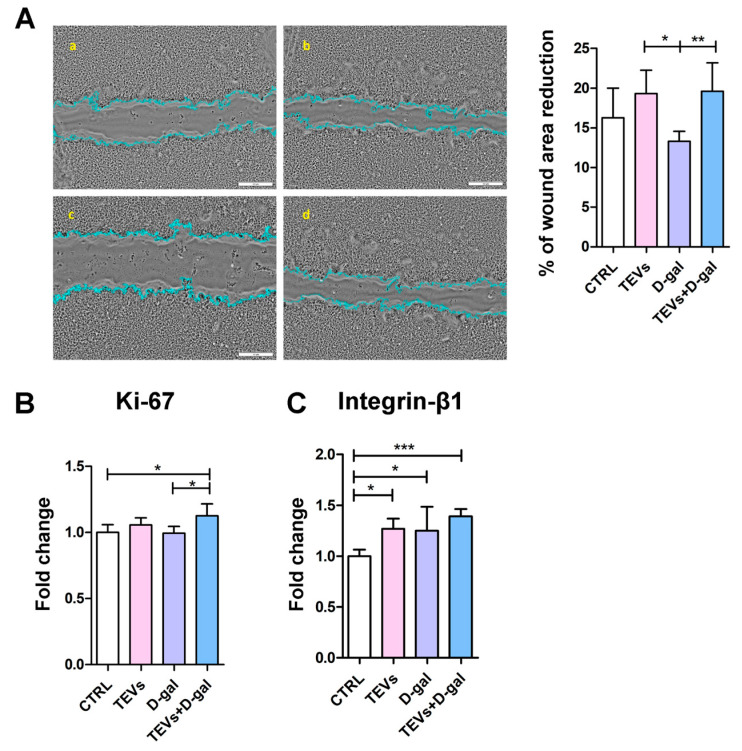
Trophoblast-derived extracellular vesicle (TEV) pretreatment enhances keratinocyte migration and Ki-67 and integrin-β1 subunit expression after D-gal exposure. (**A**) Representative phase-contrast photomicrographs ((**a**) CTRL; (**b**) TEVs; (**c**) D-gal; (**d**) TEVs+D-gal) show keratinocyte migration after 24 h. Bar graphs indicate (**A**) the percentage of wound closure relative to the initial scratch area and the protein level of (**B**) Ki-67 and (**C**) integrin-β1 subunit expression in keratinocytes, as determined by cELISA. Cumulative data from two experiments (3–5 replicates) are shown as mean fold change relative to the unexposed cells (CTRL) + SD. * *p* < 0.05; ** *p* < 0.01; *** *p* < 0.001.

To investigate the potential beneficial effects of TEV pretreatment in the given experimental setting, we conducted a wound healing assay. For this purpose, a controlled “wound” was created on a monolayer of keratinocytes using a pipette tip, and the ability of the cells to migrate and proliferate to close the wound gap was observed over 24 h. The rate of wound closure was lower in the cell cultures exposed to D-gal compared to the control cells and those treated with TEVs alone (Figure 5A). However, this difference was statistically significant only between D-gal-exposed cells and those treated with TEVs only. Notably, TEV pretreatment significantly enhanced wound closure in D-gal-exposed cells, even exceeding the control level (Figure 5A), highlighting the potential of TEVs to promote tissue repair.

Next, to examine the effect of TEV pretreatment on some of the putative mechanisms underlying “wound” closure, we analyzed the expression Ki-67, a widely used proliferation marker [50]. The protein level of Ki-67 was higher in the cells pretreated with TEVs and exposed to D-gal than in the other experimental groups (Figure 5B). This difference was statistically significant only when compared to the control cells and those exposed to D-gal only (Figure 5B).

Additionally, recognizing that senescent cells often exhibit altered adhesion properties and impaired migration due to changes in integrin expression and function [51], we measured the expression of integrin-β1 subunit, a key adhesion receptor subunit that mediates interactions between keratinocytes and the ECM, which are crucial for migration, proliferation, and tissue regeneration [52]. All treatments led to a significant increase in integrin-β1 subunit expression compared to control cells, with the most pronounced increase observed in the cells pretreated with TEVs and then exposed to D-gal (Figure 5C). The significantly increased expression of Ki-67 and integrin-β1 subunit in TEV-pretreated keratinocytes indicates TEV potential to stimulate keratinocyte proliferation and adhesion, both of which are critical for wound closure [50,53].

## 4. Discussion

The study presented herein highlights the potential of TEVs to counteract D-gal-induced premature aging in keratinocytes by reducing stress and senescence markers while enhancing regenerative capacity.

TEVs were isolated using differential ultracentrifugation and a comprehensive characterization of their physical and molecular features was performed. NTA revealed a heterogeneous population of vesicles ranging from 111 to 302 nm in diameter, with a mean size of approximately 168 nm. The multimodal particle distribution observed in our study aligns with previous reports on TEVs [26] and EVs derived from various other cell types, including mesenchymal stem cells (MSCs) [54] and cardiomyocytes [55], which also demonstrate a wide size range, indicating the heterogeneous nature of EV populations. TEM confirmed the presence of electron-dense structures consistent with EV morphology, exhibiting well-preserved membrane integrity.

Super-resolution dSTORM microscopy provided valuable single-vesicle-level insights, confirming the presence of canonical tetraspanin EV markers—CD9, CD63, and CD81—on the surface of TEVs [34]. Most vesicles were positive for at least one tetraspanin, with CD9 showing the highest prevalence (92.5%), followed by CD81 (78.9%) and CD63 (51.8%). Interestingly, 39% of the TEVs co-expressed all three tetraspanins, which suggests a subset of vesicles with a highly enriched EV marker profile. These findings are consistent with the growing recognition that tetraspanin expression is variable among EV subpopulations and may reflect differences in their biogenesis or cellular origin [56]. Namely, the relatively lower proportion of CD63+ vesicles compared to CD9+ and CD81+ ones could indicate the presence of both endosomal (CD63-enriched) and plasma membrane-derived EVs (CD9/CD81-enriched), supporting the concept of TEV heterogeneity [57,58].

D-gal is a monosaccharide widely used to model senescence, characterized by oxidative and metabolic stress, inflammation, and cellular dysfunction. Chronic exposure impairs antioxidant defenses, leading to ROS accumulation and oxidative damage [6,10]. Considering that trophoblast cells face varying levels of OS during pregnancy and have developed robust antioxidant defenses [59], it is plausible to assume that TEVs contain antioxidants or signaling molecules that could influence keratinocyte OS responses. By supplying bioactive molecules such as antioxidants, anti-inflammatory and repair proteins, growth factors, and non-coding RNAs, TEVs could also help mitigate DNA damage, promote cell survival, and preserve genomic integrity. Indeed, in our study, TEV pretreatment significantly reduced keratinocyte DNA damage after D-gal exposure, indicating a protective role for TEVs against OS-induced genomic instability, a key factor in premature senescence [6]. Furthermore, TEVs derived from EVT Swan 71 cells were shown to contain respiratory complex antioxidants such as NADH–ubiquinone oxidoreductase and peroxiredoxin-1 and 4 [19]. Also, an *in vivo* study demonstrated that TEVs ameliorated doxorubicin-induced OS in cardiomyocytes, possibly by improving mitochondrial membrane potential and mitochondrial dynamics [60]. Similarly, MSC-derived EVs mitigated oxidative injury, OS-induced aberrant calcium signaling, and mitochondrial changes in both primary culture of murine keratinocytes *in vitro* and *in vivo* in UV-irradiated murine skin [61].

The molecular analyses also showed TEV pretreatment downregulated the expression of p19 and p21—CDK inhibitors implicated in the regulation of cellular senescence and proliferation—in keratinocytes that have been exposed to D-gal. This suggests that TEVs might rescue cell cycle arrest, preventing the induction of senescence and contributing to the maintenance of keratinocyte homeostasis even under stress conditions. Notably, TEVs alone did not influence p19/p21 expression, suggesting that their protective effect is specifically related to stressed cells. To corroborate these results are data showing that the cells pretreated with TEVs showed a partial restoration of the normal cell cycle distribution, with fewer cells in G0/G1 and more progressing to the G2/M phase. These results suggest that TEVs help preserve cell cycle progression and proliferation in HaCaT cells under D-gal-induced stress. This may be associated with the TEV content of bioactive factors that promote cell survival and counteract the senescence-inducing signals triggered by D-gal. As shown previously, modulation of the p53/p21 pathway by miR-125b-5p and miRNA-214-3p from MSC-derived EVs prevented stress-induced cellular senescence, thereby enhancing cell survival [62,63]. Additionally, by countering OS, TEVs may prevent the activation of the DNA damage response that leads to senescence [6].

The accumulation of HIF-1α most likely also contributed to the observed upregulation of cell cycle inhibitors in D-gal-exposed keratinocytes [44]. Namely, HIF-1α, a stress-responsive factor involved in senescence and skin homeostasis [45,46], was upregulated in keratinocytes treated with either TEVs or D-gal alone, indicating elevated metabolic stress or adaptation. While D-gal-induced HIF-1α accumulation probably reflects OS and senescence-related response (as mitochondrial ROS can activate the MEK/ERK pathway, stabilizing HIF-1α in keratinocytes [46]), TEV-induced HIF-1α elevation may not necessarily signify harm. Instead, it may represent a preconditioning or adaptive response, where TEVs trigger mild, beneficial signaling—such as hypoxia-related or metabolic pathways—that prime cells for resilience [64,65]. This hormesis-like effect may enhance survival without inducing damage. Notably, TEV pretreatment prior to D-gal exposure significantly lowered HIF-1α levels compared to D-gal alone, suggesting TEVs help restore metabolic balance and mitigate stress-induced dysfunction. This may be associated, at least partly, with TEV antioxidative properties.

Given that HIF-1α is regulated by mTOR [47]—a key player in cell growth and aging [48]—mTOR expression was also assessed. D-gal increased mTOR expression, consistent with previous findings linking D-gal to OS and mTOR activation [42,49]. In contrast, TEVs alone reduced mTOR mRNA, and TEV pretreatment normalized mTOR levels even under D-gal challenge. Previously, MSC-derived EVs were also shown to modify mTOR expression in stressed cells by inhibiting the activation of phosphoinositide 3-kinase (PI3K)/AKT/mTOR signaling [66].

These findings suggest that TEVs do not act as stressors but rather as modulators of metabolic resilience. They may promote recovery from oxidative and metabolic stress through multiple mechanisms: activation of adaptive HIF-1α-mediated pathways supporting mitochondrial function and glycolysis [67,68], delivery of bioactive molecules that regulate antioxidant and repair responses [69], and enhancement of intercellular communication [70]. Collectively, these effects position TEVs as facilitators of cellular homeostasis and stress recovery, particularly in the context of D-gal-induced dysfunction.

Recent studies have also highlighted EV regenerative potential, particularly in promoting cell proliferation [71]. In that vein, TEVs are shown to exhibit anti-aging and regenerative properties by enhancing the proliferation of dermal fibroblasts and by modulation of signaling pathways associated with cell survival, growth, and tissue repair [72]. In this study, D-gal exposure alone impaired wound closure, indicating reduced migratory and proliferative capacity under oxidative and metabolic stress. TEV pretreatment markedly improved wound closure in D-gal-exposed cells—surpassing even control levels—suggesting a strong pro-repair effect. These results are consistent with previous studies showing that EVs from various stem/progenitor cells enhance wound healing *via* modulation of key repair pathways [73]. Proteomic analyses suggest that TEVs induce the activation of matrix metalloproteinases (MMPs) and mitogen-activated protein kinase (MAPK) signaling pathways [74], which are crucial for tissue remodeling and wound healing [75].

To explore underlying mechanisms for the observed effect on wound healing, we analyzed the expression of Ki-67, a proliferation marker [50]. Its expression was significantly elevated in the cells pretreated with TEVs and then exposed to D-gal, supporting the idea that TEVs restore or enhance proliferative capacity under stress. We also evaluated the expression of the integrin-β1 subunit, a critical adhesion molecule involved in keratinocyte migration and tissue regeneration [52]. All treatments increased integrin-β1 subunit expression, with the greatest upregulation observed in the TEV + D-gal group. This suggests that TEVs enhance not only keratinocyte proliferation but also adhesion—both essential for efficient wound healing [50,53]. Mechanistically, TEVs may exert these effects through the delivery of bioactive molecules that activate key pathways such as MMPs and MAPKs, known to facilitate tissue remodeling and repair [74,75]. Altogether, these findings indicate that TEVs promote keratinocyte regeneration by enhancing proliferation, adhesion, and migration, effectively counteracting D-gal-induced dysfunction and supporting their potential role in therapeutic skin repair. In that regard, our previous work demonstrated that TEVs contain galectins 1, 3, and 8 [22], all of which are known to modulate integrin-β1 subunit activation and associated signaling pathways involved in cellular adhesion, migration, and proliferation [23]. Summarized, the previous results support the idea that TEVs not only counteract senescence-related impairments, but also actively promote regenerative mechanisms in keratinocytes, reinforcing their therapeutic potential in wound healing and tissue repair.

In that context, TEVs could be integrated into bioengineered models of aging skin to better mimic physiological conditions and test rejuvenation strategies *in vitro*. For example, combining EVs with 3D bioprinted skin constructs or hydrogel-based scaffolds may enhance cell viability, matrix remodeling, and tissue regeneration [76], offering an advanced platform for modeling the aging skin phenotype and evaluating therapeutic interventions.

For clinical translation, however, several challenges must be addressed. Standardizing TEV isolation and ensuring batch-to-batch consistency in vesicle content, immunogenicity and bioactivity, as well as regulatory approval, remain critical hurdles. Additionally, optimizing delivery methods (whether topical, injectable, or scaffold-integrated), is essential for effective skin-targeted administration. Engineering TEVs to enhance targeting, prolong bioactivity, or deliver specific anti-aging molecules (e.g., miRNAs, antioxidants, or growth factors) could further improve their therapeutic value. Another important aspect to consider is that EV function can differ depending on the donor and recipient cell types, as EVs transport cell-specific cargo that shapes their biological activity [77]. Future research should focus on developing robust manufacturing protocols, conducting preclinical safety and efficacy studies, and exploring combinatorial strategies (e.g., TEVs with other bioactive agents or biomaterials) to fully harness their regenerative and anti-aging potential for clinical use.

Finally, the authors would like to acknowledge that although D-gal is widely used to model OS-induced senescence, its application in keratinocyte monocultures *in vitro* presents several limitations. HaCaT cells, commonly used for such studies, may not fully replicate the behavior of primary keratinocytes or the complex tissue microenvironment of skin. Morphological and functional hallmarks of senescence—such as cell enlargement, SA-β-Gal activity, or secretory phenotype [38,78]—may be less pronounced or absent in immortalized cells, especially over short exposure times. Additionally, 2D monolayer cultures lack cell–cell and cell–matrix interactions present in native skin, which can modulate senescence responses [79]. Also, EVs often exhibit a dose–response relationship, and the doses of EVs currently used for *in vitro* assays are relatively high compared to their physiological levels [77]. Therefore, while useful for preliminary mechanistic and proof-of-concept studies, simplified *in vitro* models may not capture the full spectrum of keratinocyte senescence seen *in vivo*.

## 5. Conclusions

This study provides comprehensive evidence that TEVs exert both protective and regenerative properties in keratinocytes exposed to D-gal.

TEVs were successfully isolated and characterized, revealing a heterogeneous population enriched in canonical EV markers. Functionally, TEV pretreatment mitigated D-gal-induced OS and DNA damage, reduced senescence marker expression, and rescued cell cycle progression, likely through the modulation of HIF-1α and mTOR signaling pathways. Importantly, TEVs enhanced keratinocyte proliferation, adhesion, and migration, as demonstrated by increased Ki-67 and integrin-β1 subunit expression, and accelerated wound closure. Overall, our findings highlight the therapeutic potential of TEVs as modulators of metabolic resilience and facilitators of keratinocyte regeneration, particularly in conditions associated with OS.

## Data Availability

The datasets used and/or analyzed during the current study are available from the corresponding author upon reasonable request.

## Supplementary Materials

The following supporting information can be downloaded at: https://www.mdpi.com/article/10.3390/life15060918/s1, Figure S1: Cell viability assessed by MTT assay; Figure S2: Morphology and senescence-associated beta-galactosidase (SA-β-gal) staining of HaCaT cells exposed to D-galactose.
