# Supplementary material for: Trophoblast Extracellular Vesicles as Modulators of Keratinocyte Stress Response and Senescence
Source: Life (Basel). 2025 Jun 5;15(6):918. doi: 10.3390/life15060918 (PMC12194262; doi:10.3390/life15060918)
Supplement: Supplementary file 1 [file life-15-00918-s001.zip › life-3548941-supplementary/Supplementary figure legends.pdf]

Supplementary Figure 1. Cell viability assessed by MTT assay. Bar graphs show the viability of HaCaT keratinocytes in complete culture medium only (CTRL), exposed to trophoblast-derived extracellular vesicles (TEVs; 50 µg/ml TEV protein), 20, 40, 60 and 80 mg/ml D-galactose (D-gal) for 24 h, as determined by the MTT assay. Cumulative data from two experiments are shown as mean % change relative to the unexposed cells (CTRL) + SD.

Supplementary Figure 2: Morphology and senescence-associated beta-galactosidase (SA-β-gal) staining of HaCaT cells exposed to D-galactose (D-gal). Photomicrographs show HaCaT cells cultivated in culture medium only or with D-gal for 48 h, and stained for SA-β-gal.
