# Supplementary figures and images for: Trophoblast Extracellular Vesicles as Modulators of Keratinocyte Stress Response and Senescence
Source: Life (Basel). 2025 Jun 5;15(6):918. doi: 10.3390/life15060918 (PMC12194262; doi:10.3390/life15060918)

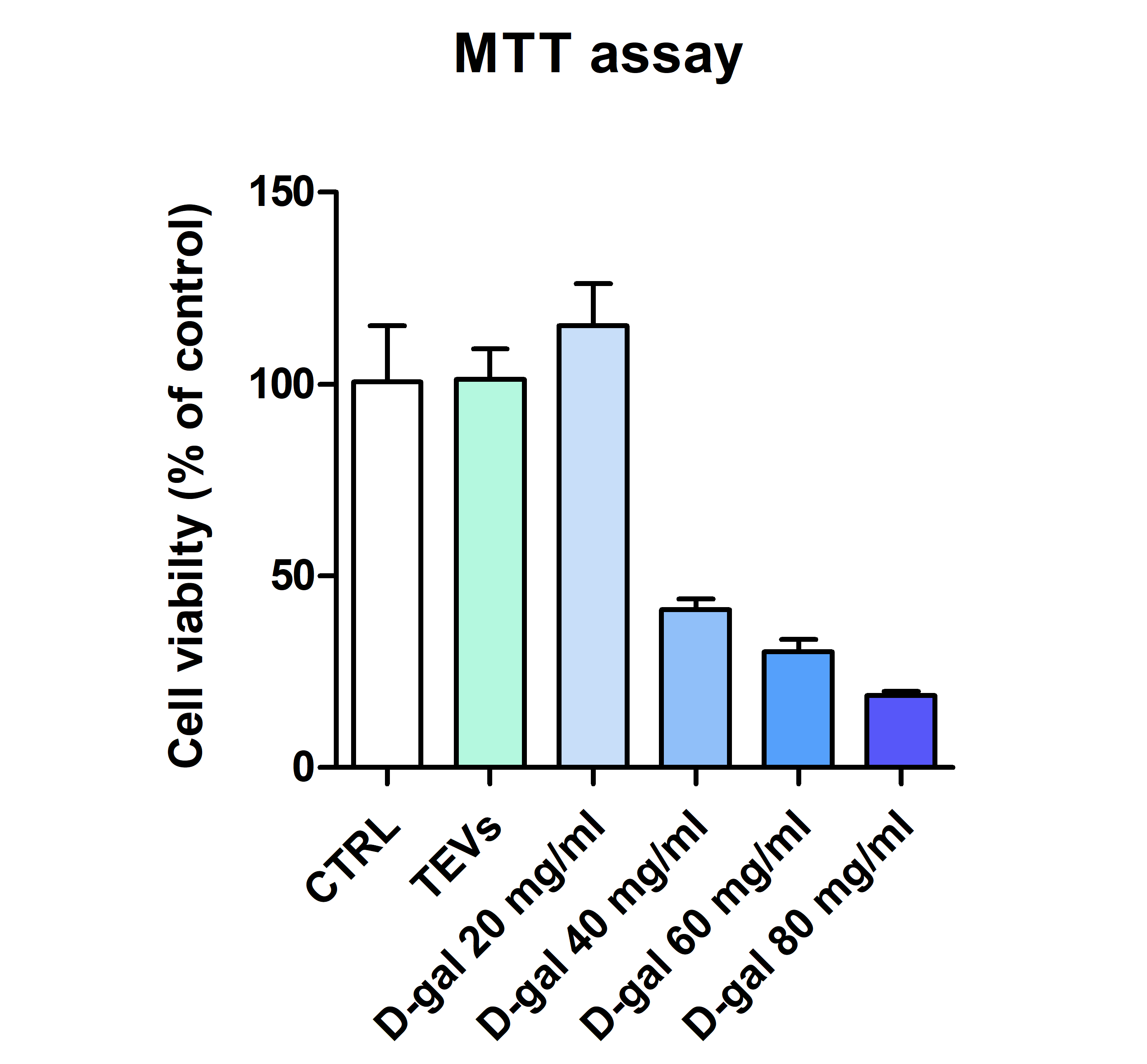

Supplement: Supplementary file 1 [file life-15-00918-s001.zip › life-3548941-supplementary/Supplementary Fig.1.tif]

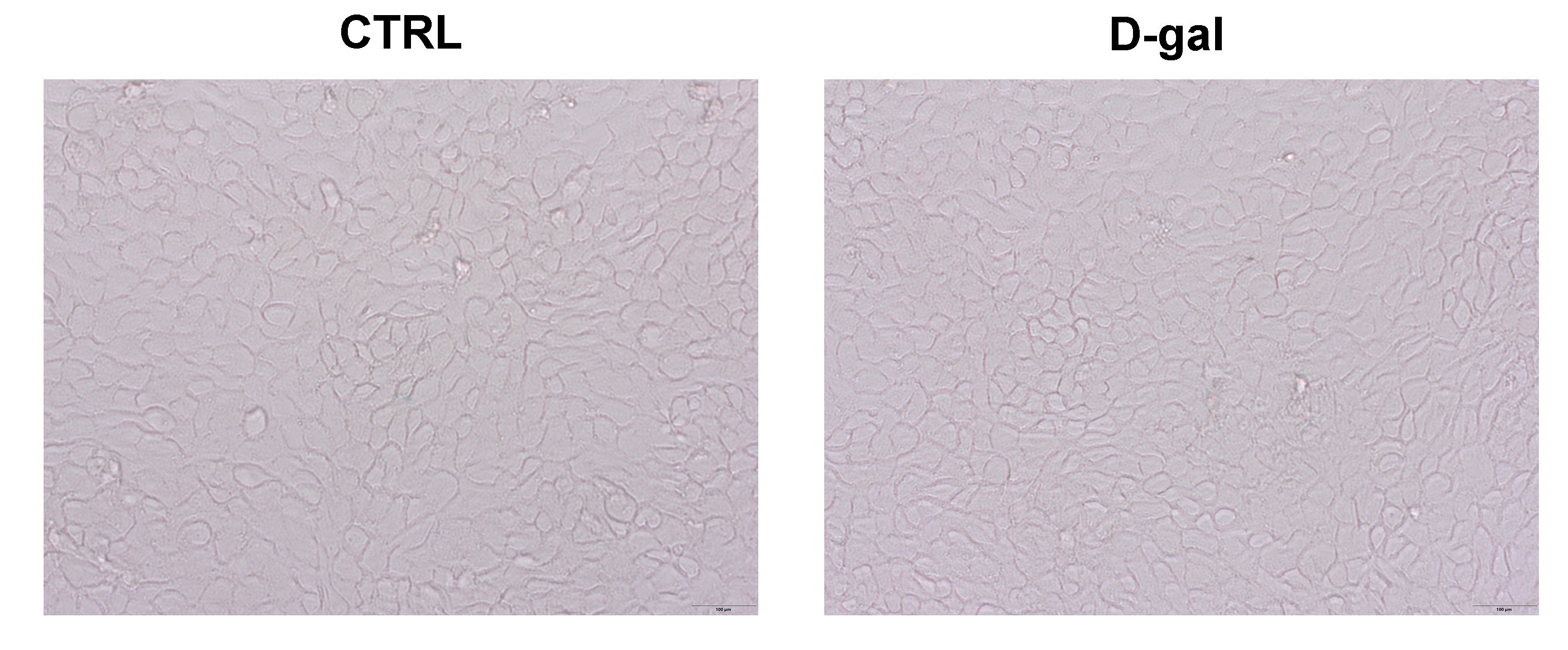

Supplement: Supplementary file 1 [file life-15-00918-s001.zip › life-3548941-supplementary/Supplementary Fig.2.tif]
